# iRGD-guided tamoxifen polymersomes inhibit estrogen receptor transcriptional activity and decrease the number of breast cancer cells with self-renewing capacity

**DOI:** 10.1186/s12951-019-0553-4

**Published:** 2019-12-07

**Authors:** María Inés Diaz Bessone, Lorena Simón-Gracia, Pablo Scodeller, María de los Angeles Ramirez, María Amparo Lago Huvelle, Galo J. A. A. Soler-Illia, Marina Simian

**Affiliations:** 1 Instituto de Nanosistemas, Universidad Nacional de San Martín, 25 de Mayo 1021, San Martín, 1650 Buenos Aires, Argentina; 2 Laboratory of Cancer Biology, Institute of Biomedicine and Translational Medicine, University of Tartu, Ravila 14b, 50411 Tartu, Estonia

**Keywords:** Breast cancer, Tamoxifen, Endocrine resistance, Fibronectin, iRGD-guided polymersomes, Self-renewing capacity

## Abstract

**Background:**

Tamoxifen (Tam) is the most frequent treatment for estrogen receptor (ER) positive breast cancer. We recently showed that fibronectin (FN) leads to Tam resistance and selection of breast cancer stem cells. With the aim of developing a nanoformulation that would simultaneously tackle ER and FN/β1 integrin interactions, we designed polyethylene glycol-polycaprolactone polymersomes polymersomes (PS) that carry Tam and are functionalized with the tumor-penetrating iRGD peptide (iRGD-PS-Tam).

**Results:**

Polyethylene glycol-polycaprolactone PS were assembled and loaded with Tam using the hydration film method. The loading of encapsulated Tam, measured by UPLC, was 2.4 ± 0.5 mol Tam/mol polymer. Physicochemical characterization of the PS demonstrated that iRGD functionalization had no effect on morphology, and a minimal effect on the PS size and polydispersity (176 nm and Pdi 0.37 for iRGD-TAM-PS and 171 nm and Pdi 0.36 for TAM-PS). iRGD-PS-Tam were taken up by ER+ breast carcinoma cells in 2D-culture and exhibited increased penetration of 3D-spheroids. Treatment with iRGD-PS-Tam inhibited proliferation and sensitized cells cultured on FN to Tam. Mechanistically, treatment with iRGD-PS-Tam resulted in inhibition ER transcriptional activity as evaluated by a luciferase reporter assay. iRGD-PS-Tam reduced the number of cells with self-renewing capacity, a characteristic of breast cancer stem cells. In vivo, systemic iRGD-PS-Tam showed selective accumulation at the tumor site.

**Conclusions:**

Our study suggests iRGD-guided delivery of PS-Tam as a potential novel therapeutic strategy for the management of breast tumors that express high levels of FN. Future studies in pre-clinical in vivo models are warranted.

## Introduction

Breast cancer affects one in eight women worldwide [1]. Seventy five percent of diagnosed breast cancers are estrogen receptor (ER) positive [2]. Tamoxifen (Tam), a selective ER modulator, is the main endocrine treatment prescribed to breast cancer patients [3], and current guidelines recommend 10 years of treatment [4]. One third of patients receiving Tam will relapse within 15 years of their initial diagnosis. Tam resistance is traditionally associated with overexpression/activation of growth factor receptors such as HER2, or IGF-R1 [5]. However, only 20% of patients present alterations in these signaling pathways suggesting that other mechanisms are involved. We have demonstrated that binding of β1 integrins to fibronectin (FN) confers tamoxifen resistance to otherwise sensitive breast cancer cells [6]. In clinical human breast cancer samples, high expression of extracellular matrix components (such as FN, collagen 1A1, tenascin-C), and their receptors (e.g. β1 integrins) is associated with endocrine resistance [7, 8]. Moreover, both in vitro and in vivo, Tam exposure leads to the selection of β1-positive breast cancer cells with self-renewing capacity, a characteristic of breast cancer stem cells [9, 10]. Additionally, we recently demonstrated that β1-integrin co-localizes with ERα at the cell membrane and in endosomes in breast cancer cell lines and in human normal and neoplastic tissue samples [11]. In the presence of FN, β1 prolongs ERα’s half-life and strengthens its transcriptional activity [11]. We hypothesized that targeting of β1 integrin/FN interaction in combination with ER blockade could be an effective strategy to avoid emergence of FN-induced Tam resistance and the selection of breast cancer stem cells—events associated with cancer recurrence.

Nanoparticles (NPs) have physicochemical and biological properties that are fundamentally different from those that characterize the individual components that make them up. The ability to carry different hydrophilic and hydrophobic imaging and therapeutic payloads, their cellular uptake by endocytosis, and possibility of engineering multiple functions into single nanocarrier system (multifunctionality) render NPs interesting for drug delivery and diagnosis. Compared to free drugs, NP-based agents can be engineered to have improved biodistribution, pharmacokinetics, and toxicological properties. Polymersomes (PS) are formed by the self-assembly of amphiphilic copolymers in aqueous solutions into nanoscale vesicles that can accommodate hydrophilic and hydrophobic drugs at high concentrations [12–14] and be labeled with different tracers: optical probes, MRI, and PET imaging agents [15]. PS exhibit an intrinsic tumor tropism that can be further enhanced by functionalization with affinity ligands for precision-guided delivery to solid malignancies [16, 17]. Among them, the iRGD peptide (amino acid sequence: CRGDKGPDC) belongs to a new class of targeting ligands, tumor-penetrating peptides, that home to tumors and are actively transported into the extravascular tumor parenchyma [18]. iRGD contains an αv-integrin-binding motif and an RGDK cryptic CendR motif that upon proteolytic activation mediates binding to neuropilin-1 (NRP-1) that is overexpressed and correlates with increased aggressiveness in several solid tumor types, including breast cancer [18, 19]. When conjugated to therapeutic PS (and to other types of NPs), iRGD improves their tumor homing, penetration, and antitumor efficacy [18, 20, 21]. Moreover, iRGD has been shown to potentiate the effect of several drugs when co-administered with them [22] and is currently clinically developed for combination therapy of pancreatic cancer with gemcitabine and albumin-paclitaxel NPs (ClinicalTrials.gov identifier: NCT03517176).

At the cellular level, iRGD was previously shown to dramatically collapse cellular protrusions on FN-coated surfaces through a neuropilin-1 (NRP-1)-mediated negative regulation of FN-binding β1-integrins [23]. This effect appeared to be specific for FN, as attachment of the cells to collagen I, mainly recognized by α1β1 and α2β1 integrins, was not inhibited by iRGD [23]. We hypothesized that the application of iRGD-functionalized Tam-loaded PS could be a rational strategy to reduce the selection of breast cancer stem cells by Tam and counteract the induction of endocrine resistance by FN.

Here we investigated the impact of iRGD-functionalized Tam-loaded PS on cultured human ER+ breast cancer cells and found that they resensitize cells to Tam when these are cultured in the presence of FN. Moreover, enrichment of breast cancer cells with self-renewing capacity induced by Tam is inhibited in the presence of iRGD. iRGD-functionalized Tam-loaded PS effectively inhibited ER’s transcriptional activity as determined by a transcription reporter assay. Finally, we demonstrate that targeted PS home to breast cancer xenograft lesions in mice, suggesting potential therapeutic applications for in vivo modulation of ER-dependent cancers.

## Results

### Assembly and characterization of Tam-loaded PS

**Fig. 1:**
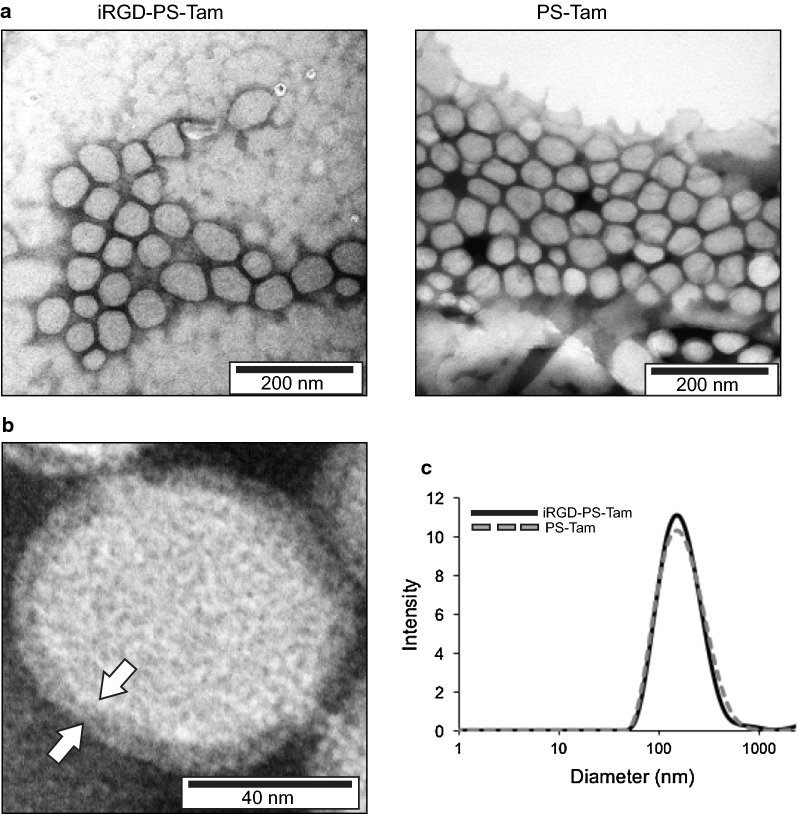
Characterization of Tam-loaded PS. **a** TEM images of Tam-loaded iRGD-functionalized and untargeted PS. **b** TEM image of Tam-loaded PS showing the vesicle membrane (white arrows). **c** Size distribution measured by DLS of the iRGD-PS-Tam and PS-Tam

The PS were assembled and loaded with Tam using the hydration film method [24]. The loading of encapsulated Tam, measured by UPLC, was 2.4 ± 0.5 mol Tam/mol polymer. iRGD PS labelling was determined by fluorescence. A calibration curve was performed with different concentrations of free iRGD, and iRGD concentration (0.026 ± 0.0025 mg/mL) was obtained by linear regression (Additional file 1: Figure S1). TEM analysis demonstrated that iRGD functionalization had no effect on PS morphology (Fig. 1a) and confirmed the vesicular structure of the PS membrane (Fig. 1b, arrows). DLS analysis demonstrated that iRGD functionalization had a minimal effect on the PS size and polydispersity (176 nm and Pdi 0.37 for iRGD-TAM-PS and 171 nm and Pdi 0.36 for TAM-PS; Fig. 1c).

### iRGD-functionalization increases uptake of Tam-loaded PS in cultured breast cancer cells

**Fig. 2:**
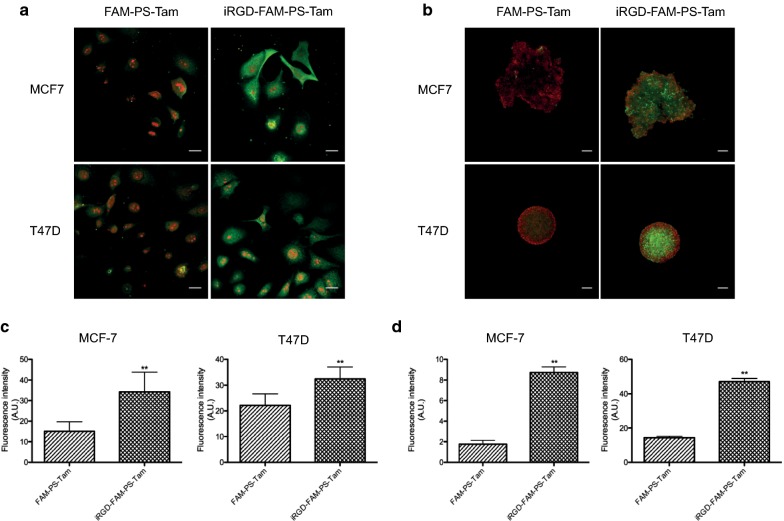
Internalization of PS in breast cancer cells in 2D and 3D culture. **a** MCF7 and T47D cells were seeded on glass coverslips in 24-well plates. After 24 h, FAM-PS-Tam or iRGD-FAM-PS-Tam were added to the cells at a concentration of 0.5 mg/mL and incubated for 1 h or 24 h. Confocal images of cells at 24 h of treatment. PS are labelled in green and nuclei are counterstained with propidium iodide (red). Scale bars: 20 μm. **b** MCF7 and T47D spheroids were allowed to develop for 7 days as explained in materials and methods. At that time, they were either treated with FAM-PS-Tam or iRGD-FAM-PS-Tam at a concentration of 0.5 mg/mL and incubated for 1 h or 24 h. Confocal images at 24 h are shown. PM are labelled in green and nuclei are counterstained with propidium iodide (red). Scale bars: 100 μm. **c** Quantification of the fluorescence intensity/cell for MCF-7 and T47D cells. A statistically significant increase was detected in cells treated with iRGD-FAM-PS-Tam compared to FAM-PS-Tam; graph shows PS fluorescence in arbitrary units; bars represent mean ± SEM; N = 3; student’s *t* test was performed to analyze statistical significance, **p < 0.01. **d** Quantification of the fluorescent intensity per spheroid for MCF-7 and T47D cells. A statistically significant increase was detected in spheroids treated with iRGD-FAM-PS-Tam compared to FAM-PS-Tam. Graph shows PS fluorescence in arbitrary units; bars represent mean ± SEM; N = 3; student’s *t* test was performed to analyze statistical significance **p < 0.01

We used MCF7 [25] and T47D [26] human breast cancer cell lines that are ER positive, sensitive to Tam and express NRP-1 [27] to study the interactions of FAM-labeled iRGD-guided and control PS with the cultured breast cancer cells. In 2D culture, functionalization of FAM-PS-Tam with iRGD resulted in a significant increase in the uptake of the PS in both cell lines, as determined by confocal microscopy (Fig. 2a) and quantification of the fluorescence intensity per cell (Fig. 2c).

Compared to 2D culture, cells in 3D culture represent increased physiological relevance [28]. As previously described, MCF-7 and T47D cells produce mass-like spheroids that are characterized by disorganized nuclei and robust cell–cell adhesion [29]. MCF-7 spheroids were more irregular and less compact than those generated by T47D cells. This may be explained by the higher levels of E-cadherin expressed by T47D cells in comparison to MCF7 cells [30]. After developing for 7 days, spheroids were treated with iRGD-FAM-PS-Tam or FAM-PS-Tam at 0.5 mg/mL for 24 h. Irrespective of the morphology, FAM-labeled iRGD-PS-Tam were found distributed across both MCF7 and T47D spheroids, including the central cores, where high levels of fluorescence were observed (Fig. 2b, d). The control, non-targeted particles showed lower binding and were located mostly on the surface of the spheroids. Thus, derivatizing PS with iRGD significantly contributed to the uptake of the vehicles both in 2D and 3D culture models and increased their penetration in the 3D cultures.

### iRGD-PS-Tam are cytotoxic on cultured breast cancer cells

**Fig. 3:**
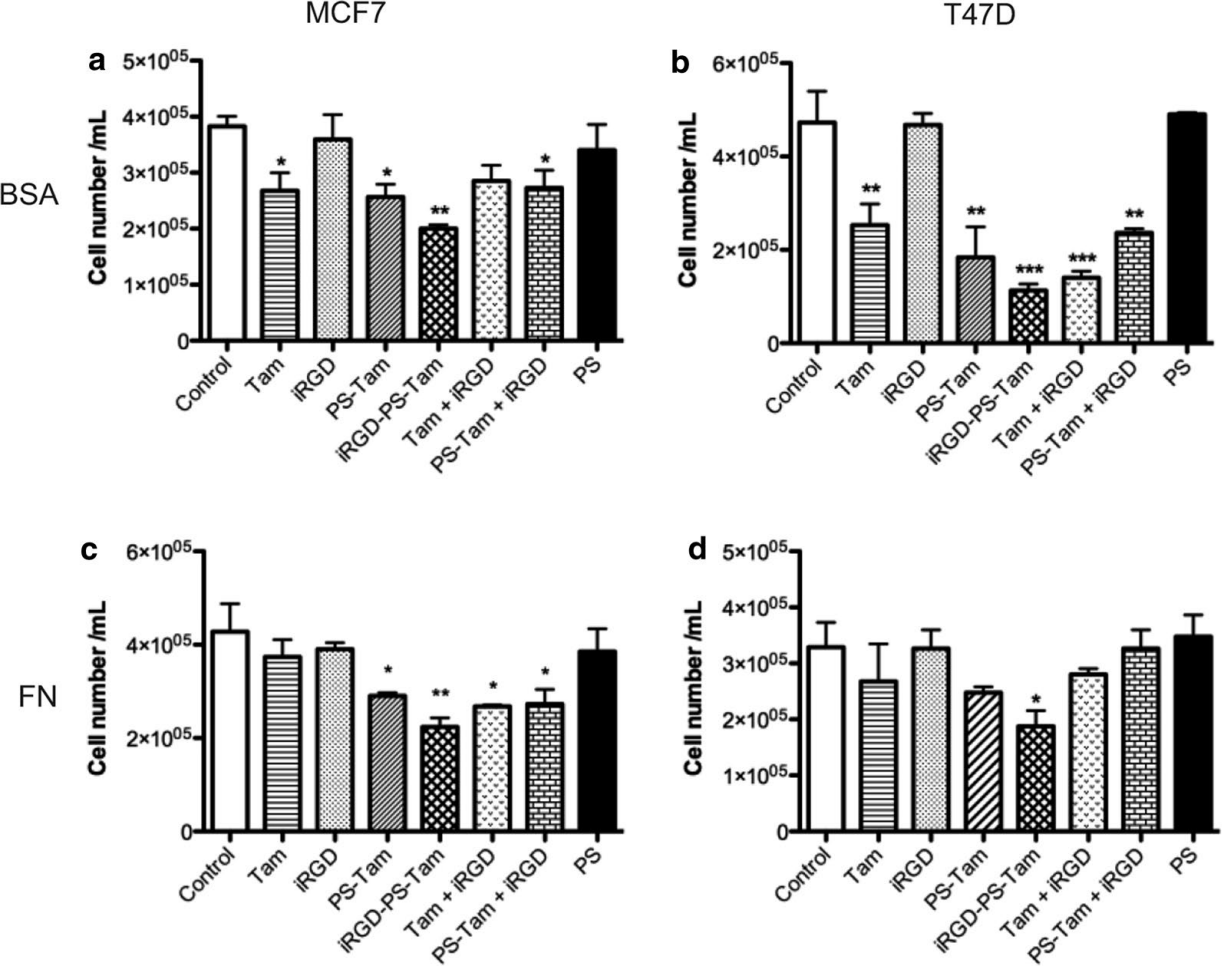
Impact of iRGD-PS-Tam and control compounds on cell viability. Sixty thousand MCF7 or T47D cells were plated in 24 well plates coated with BSA or FN. Cells were treated for 96 h with Tam, iRGD, PS-Tam, iRGD-PS-Tam, Tam + iRGD, PS-Tam + iRGD or empty PS (PS). **a**, **b** Impact of the treatments on MCF7 and T47D cells plated on BSA. Bars represent mean ± SEM; N = 3; *p < 0.05;**p < 0.01; one-way Anova followed by Sidak’s multiple comparisons was used to compare groups, ***p < 0.001, N = 3. **c**, **d** show the effect of the treatments on MCF7 and T47D cells plated on FN. Bars represent mean ± SEM; N = 3; one-way Anova followed by Bonferroni–Sidak’s multiple comparisons was used to compare the groups *p < 0.05;**p < 0.01; ***p < 0.001

MCF7 and T47D cells are sensitive to anti-estrogens, and Tam encapsulation in nanovehicles can be used to increase its toxicity [31, 32]. Moreover, to date Tam has not been encapsulated in PS and additionally combined with iRGD. We next studied the effect of treatment with iRGD-PS-Tam and control compounds (at equivalent concentrations, as detailed in “Materials and methods”) on MCF7 and T47D breast cancer cell lines cultured on BSA and FN matrices (Fig. 3). When MCF7 and T47D cells were cultured on BSA, iRGD-PS-Tam affected cell viability to a greater extent than free Tam (Fig. 3a, b). For both cell lines, encapsulation of Tam in PS did not increase the cytotoxic effect of free Tam (Fig. 3a, b). In the case of T47D cells the co-exposure to iRGD increased the cytotoxic effect of free Tam (Fig. 3b). When the cells were cultured on FN, free Tam did not have a significant effect on cell viability, as previously shown [6] (Fig. 3c, d). Interestingly, in MCF7 cells PS-Tam reverted this effect. Importantly, treatment of cells with iRGD-PS-Tam significantly decreased cell viability in both MCF7 and T47D cell lines (Fig. 3c, d). For MCF7 cells, co-administration of iRGD with either PS-Tam or free Tam also resensitized the cells to the anti-estrogen when cultured on FN. These results suggest that the encapsulation of Tam into PS partially increases the effectiveness of Tam treatment (at least in MCF7 cells), and that the incorporation of iRGD coating of the Tam-loaded PS increases the cytotoxic effect and reverts resistance induced by FN in both cell lines.

### iRGD-PS-Tam effectively inhibit ER transcriptional activity

**Fig. 4:**
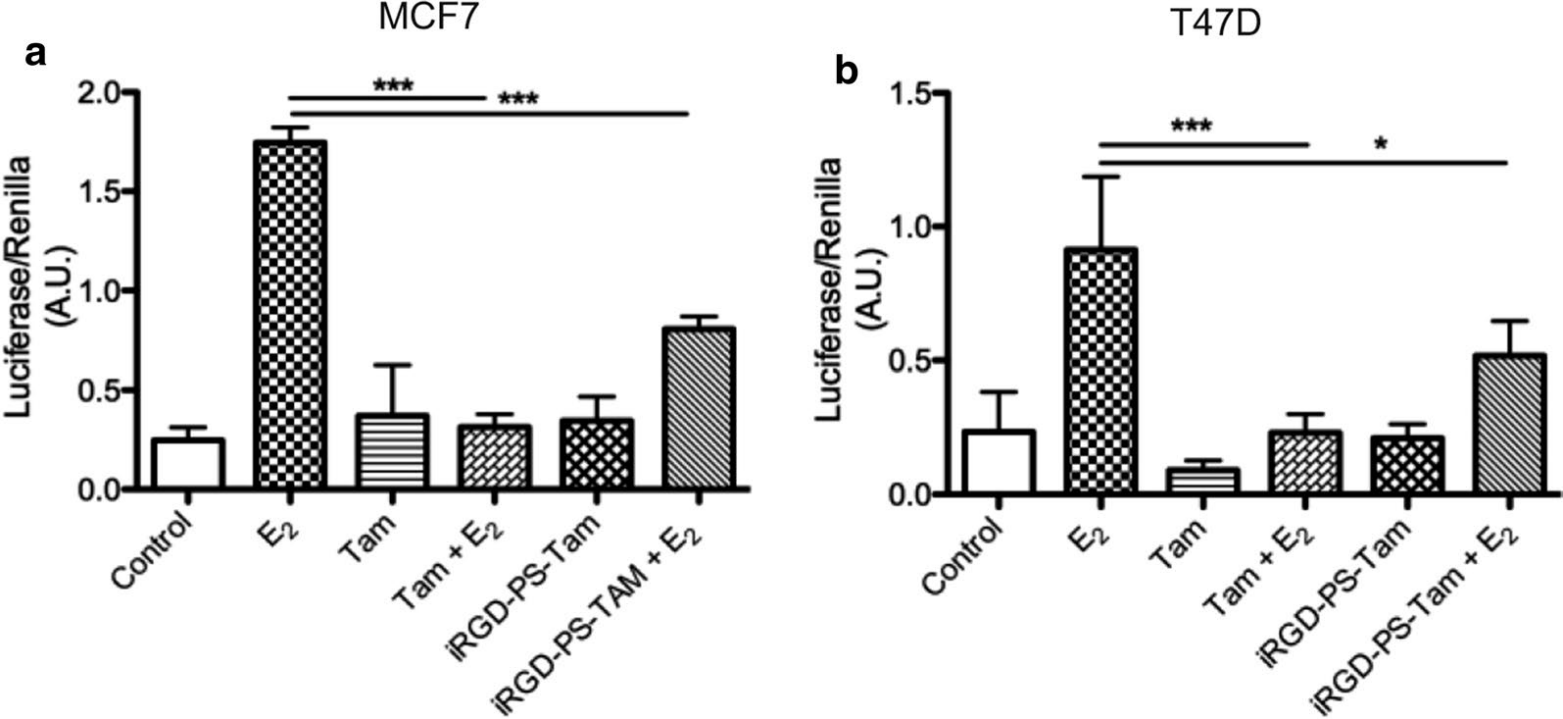
iRGD-PS-Tam inhibits ER transcriptional activity. MCF7 (**a**) and T47D (**b**) cells were plated in 48 well plates and transiently transfected with PTK-ERE-Luc and pTK Renilla reporter constructs. The cells were subsequently treated for 24 h with estradiol (E_2_), Tam, iRGD-PS-Tam, E_2_ + Tam and E2 + iRGD-PS-Tam. Data are represented as mean ± SD, N = 3, one-way Anova followed by Bonferroni–Sidak’s multiple comparisons was used to compare groups *p < 0.05; ***p < 0.001

Tam is a selective ER modulator that competitively binds to ER and inhibits its transcriptional activity [3]. In Tam-sensitive cells this inhibition is associated with cell cycle arrest and apoptosis [3]. An inhibitory effect of Tam-loaded nanovehicles on transcriptional activity of ER has not been reported. To confirm that iRGD-PS-Tam can effectively release Tam to inhibit ER transcriptional activity, we carried out transcription reporter assays. MCF7 and T47D cells were transiently transfected with an estrogen-response-element (ERE)-luciferase construct together with a tyrosine-kinase (TK)-Renilla plasmid and treated with estradiol, in the presence and absence of Tam and iRGD-PS-Tam. As expected, exposure to free Tam dramatically inhibited the estradiol-induced transcriptional activity of ER in both cell lines (Fig. 4a, b). A similar degree of inhibition was observed when cells were treated with estradiol in the presence of iRGD-PS-Tam. Interestingly, the inhibition of transcriptional activity with the iRGD-PS-Tam became apparent only at 20 h, and not at 8 h—time point usually used for experiments with free Tam (not shown). These results show that iRGD-PS-Tam exposure results in sustained intracellular release of Tam in its active form and is effective in inhibiting ER’s transcriptional activity.

### Treatment with iRGD-PS-Tam reduces the number of cancer cells with self-renewing capacity

**Fig. 5:**
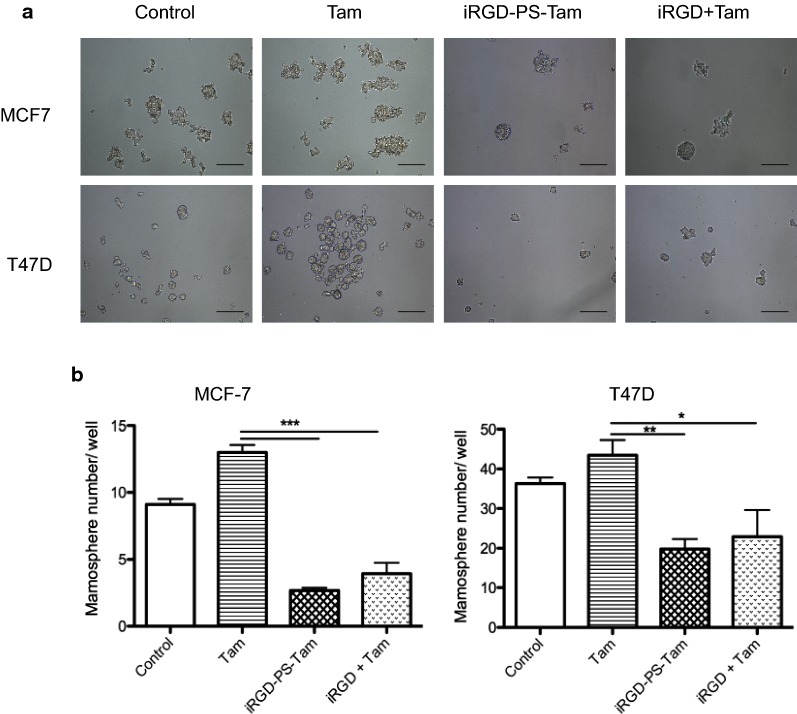
iRGD-PS-Tam exposure reduces the number of tumorspheres. MCF7 and T47D cells were pre-treated for 48 h with vehicle (control), Tam, iRGD-PS-TAM or iRGD + Tam and then plated in non-adherent plates under tumorsphere culture conditions as explained in materials and methods. **a** Representative images of tumorspheres of MCF-7 and T47D generated from pre-treated cells as explained. Scale bars: 200 μm. **b** Tumorsphere count per well is shown for MCF7 and T47D cells. Data are represented as mean ± SD, N = 3, one-way Anova followed by Bonferroni–Sidak’s multiple comparisons was used to compare groups *p < 0.05; ***p < 0.001

Previously, we and others showed that Tam leads to the positive selection of cells with self-renewing capacity, a characteristic associated to breast cancer stem cells [9, 10, 33]. Moreover, these cells in breast cancer are characterized by the high expression of the β1 integrin subunit (CD29) [34]. Thus, we explored whether the presence of iRGD on Tam-loaded PS would interfere with the enrichment in breast cancer cells with self-renewing capacity induced by Tam. 2D-cultured MCF7 and T47D cells were treated with free Tam, iRGD-PS-Tam, and iRGD in combination with free Tam for 72 h. Next, the cells were detached and re-plated on non-adherent cell culture plates in serum-free medium containing EGF and B27—conditions known to promote the development of breast cancer tumorspheres [35]. As shown in Fig. 5a, b, exposure to free Tam resulted in increased formation of tumorspheres. In contrast, for both MCF7 and T47D cell lines, the treatment with iRGD-PS-Tam reduced the number of cells capable of developing spheres. Interestingly, treatment of cells with iRGD in combination with free Tam had the same effect, suggesting that blocking β1-integrins with this peptide may be sufficient to reduce breast cancer stem cells. These results have important clinical implications in the neoadjuvant setting where endocrine treatment has been shown to lead to an increase in breast cancer stem cells [36].

### iRGD-PS-Tam home to mouse mammary xenograft lesions

**Fig. 6:**
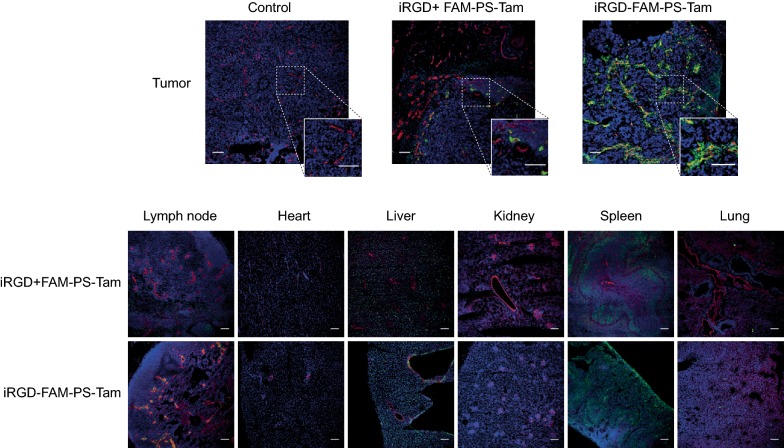
Homing of iRGD-PS-Tam to mouse mammary xenographs. Mice bearing s.c. MCF7 tumors were i.v. injected with iRGD + Fam-PS-Tam or iRGD-FAM-PS-Tam. After 4 h the animals were sacrificed and tumors and organs were collected and snap-frozen. Immunofluorescence staining with anti-FAM and CD31 primary antibodies was performed. Nuclei were counterstained with DAPI. Representative images tumors and organs from two independent experiments are shown. Scale bars: 100 μm

Next, MCF7 cells were injected in the right flank of athymic female Balb/c mice. Two weeks after tumor implantation, the mice were injected intravenously with iRGD-FAM-PS-Tam or iRGD + FAM-PS-Tam. Four hours later, the mice were sacrificed and tissues collected. Figure 6 shows immunofluorescence images of frozen tissue sections stained for CD31 in red and anti FAM in green. iRGD-FAM-PS-Tam accumulated preferentially in the tumor, as compared to other organs, showing co-localization with blood vessels positive for CD31. Non-targeted PS that were inoculated simultaneously with free iRGD showed very little accumulation in the tumor tissue. These results show that iRGD when attached to PS contributes to the homing of tamoxifen nanovehicles to the tumor tissue site (Fig. 6).

### iRGD-PS-Tam do not accumulate in normal mammary epithelial tissue adjacent to the tumor site

**Fig. 7:**
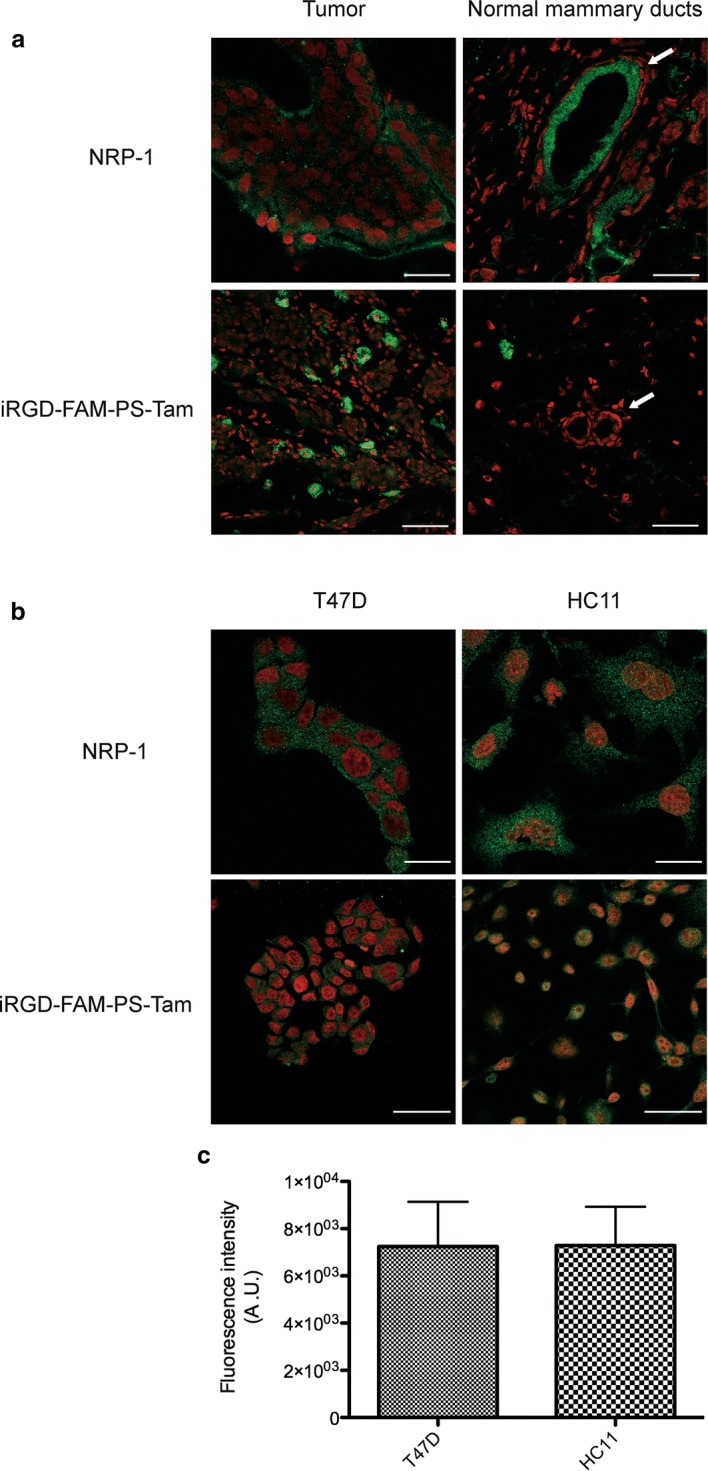
iRGD-PS-Tam do not accumulate in normal mammary epithelial tissue adjacent to the tumor site. **a** Mice bearing s.c. M05 tumors were i.v. injected with iRGD-FAM-PS-Tam. After 4 h the animals were sacrificed and tumors and adjacent mammary gland were collected and snap-frozen. Immunofluorescence staining with anti-NRP-1 and FAM primary antibodies was performed. Nuclei were counterstained with RedDot. Representative images of tumors and normal mammary gland from two independent experiments are shown, where nuclei are shown in red and specific staining in green. Scale bars: 20 μm. **b** T47D and HC11 cells were seeded on glass coverslips in 24-well plates. After 24 h, iRGD-FAM-PS-Tam were added to the cells at a concentration of 0.5 mg/mL and incubated for 1 h. PS are labelled in green and nuclei are counterstained with RedDot (red). Scale bars: 50 μm. Quantification of the fluorescence intensity/cell for T47D and HC11 cells. No statistically significant difference was detected between both cell lines; graph shows PS fluorescence in arbitrary units; bars represent mean ± SEM; N = 3; student’s t test was performed to analyze statistical significance

Normal luminal breast epithelial cells have been shown express NRP-1 [37]. Thus, we investigated whether iRGD-FAM-PS-Tam accumulated in normal tissue, adjacent to growing breast tumors. To do so we used the previously characterized M05 mouse mammary tumor that grows in immune-competent Balb/c mice [38]. In this case we chose a mouse model of breast cancer to avoid any differences in binding of iRGD between mouse and human cells in the same tissue. Mice were inoculated with M05 cells and tumors were allowed to grow for 4 weeks. At that stage, mice were treated with iRGD-FAM-PS-Tam for 4 h, as explained in materials and methods. Tumors were harvested and quick-frozen together with the adjacent normal tissue. Frozen sections of the tumors and adjacent normal mammary gland were stained for NRP-1 and iRGD-FAM-PS-Tam. As shown in Fig. 7a, expression of NRP-1 was detected in normal mammary ducts and in the tumor cells. However, iRGD-FAM-PS-Tam was only detected associated to the tumor tissue (Fig. 7a, lower panel). Additionally, we tested whether normal mouse mammary cells would, in culture, uptake iRGD-FAM-PS-Tam. To do so we used HC11 mouse mammary cells, that retain the ability to differentiate and produce milk proteins in culture [39]. First, we evaluated the levels of NRP-1, and found levels of expression similar to those of T47D cells (Fig. 7b). Next, cells were cultured in the presence of iRGD functionalized PS. Confocal microscopy images and fluorescence quantification showed that the uptake in tumor and normal mammary cells was similar in culture (Fig. 7c). These results suggest that in vivo the fact that normal mammary cells do not uptake the iRGD-coated PS is related to their accumulation at the tumor site and not in the surrounding normal tissue. This is consistent with the fact that iRGD has been shown to bind preferentially to the tumor vasculature and then penetrate the tumor tissue [40].

## Discussion

**Fig. 8:**
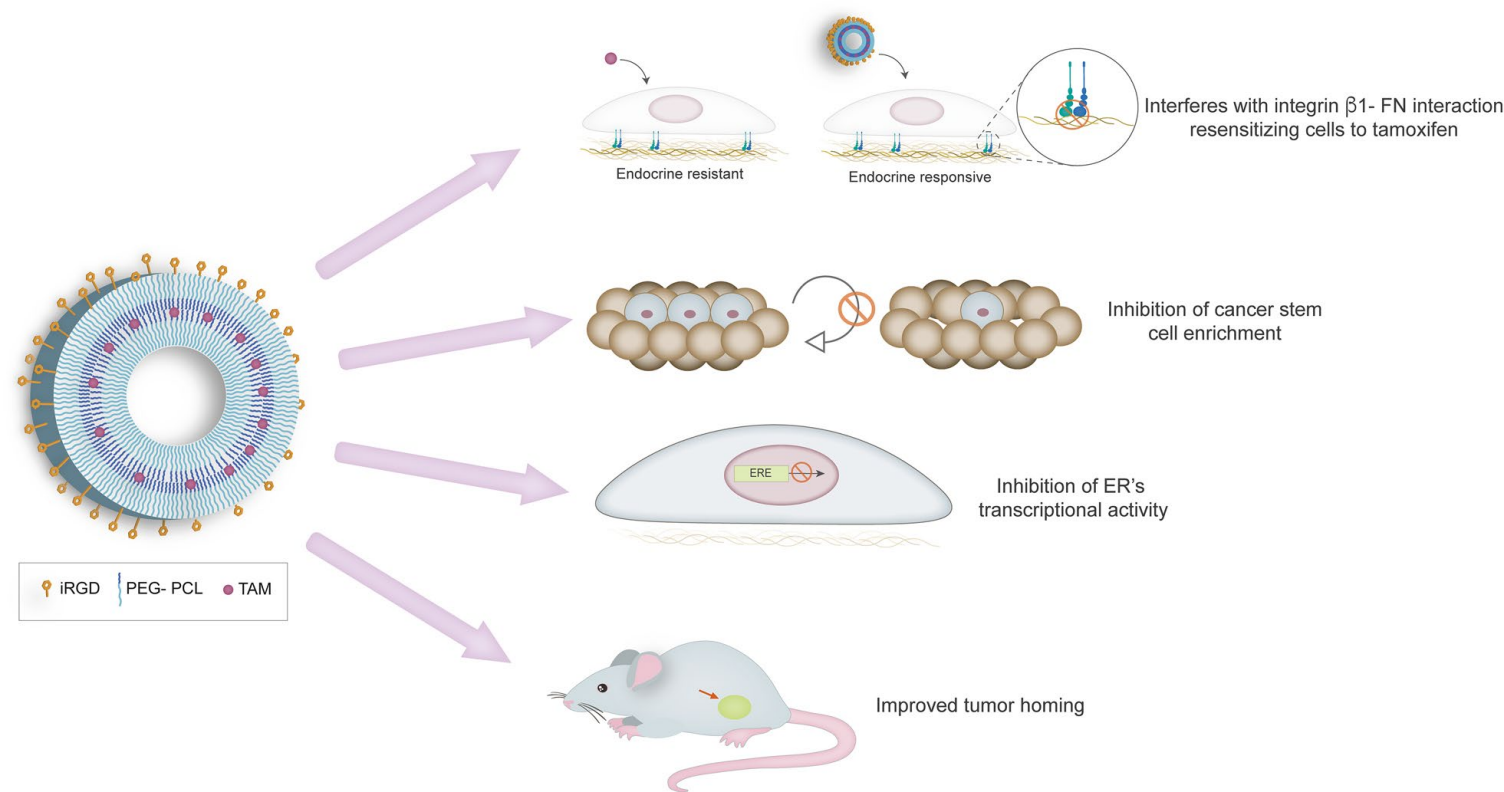
Graphical abstract. Tamoxifen (Tam) loaded, iRGD functionalized PEG-PCL polymersomes (iRGD-PS-Tam) were developed for the treatment of estrogen receptor (ER) positive breast cancer. Results show that iRGD-PS-Tam were effective in inhibiting cell proliferation and resensitizing cells cultured on fibronectin (FN) to Tam. Additionally, iRGD-PS-Tam reduced the number of cells with self-renewing capacity, a hallmark of cancer stem cells. Mechanistically, treatment of cells with iRGD-PS-TAM resulted in inhibition of ER’s transcriptional activity. Finally, in vivo studies showed selective accumulation at the tumor site

In this study, we developed and tested, on two human ER+ breast cancer cell lines, Tam-carrying PS functionalized with the tumor penetrating iRGD peptide. Our results show that Tam, delivered by these nanovehicles, is taken up by cells grown in 2 and 3D culture systems. Viability assays revealed that iRGD-PS-Tam inhibit cell proliferation and restore sensitivity to Tam when cells are grown on a FN-rich matrix. Moreover, inhibition of ER’s transcriptional activity is achieved by treatment with iRGD-PS-Tam, indicating that Tam is actually released and is active within the cell nucleus. Importantly, treatment with iRGD-PS-Tam resulted in a reduction of breast cancer cells with self-renewing capacity—a functional characteristic of breast cancer stem cells. In addition, homing of systemic PS as evaluated in vivo, and selective accumulation at the tumor site was observed for iRGD-guided PS, specifically in breast cancer cells and not in the adjacent normal tissue. These results are summarized in Fig. 8.

Tam is the main therapy received by 75% of breast cancer patients worldwide [3]. It is administered daily as a 10–20 mg oral tablet. Multiple side-effects are associated to long-term administration of Tam. In particular, Tam increases the risk of endometrial polyps and cancer, ovarian cysts, thromboembolism, thrombophlebitis, risk of stroke and ischemic attack [41]. Thus, it is of great clinical interest to develop Tam formulations that act specifically at the tumor site, without affecting other organs. Most efforts to vehiculize Tam so far have been carried out using non-targeted nanovehicles, and to our knowledge, very few biodistribution assays have been reported. For example, Martinez and collaborators developed alginate–cysteine/disulfide bond reduced albumin NPs that showed increased in vivo anti-tumor activity with increased drug concentration at the tumor site and undetectable levels of Tam in plasma [42]. They then functionalized these NPs with folate and found that this led to an increased accumulation of Tam in the tumor [43]. However, the accumulation of Tam in other organs remained the same and therapeutic response was not significantly improved as compared to the non-targeted NPs [43]. In another study, Tam was vehiculized in alpha-lipoic acid–stearylamine conjugate-based solid lipid NPs [44]. In this case bioavailability was increased and hepatotoxicity was partially decreased. However, body weight loss was still observed in treated rats as compared to controls. Dhaundiyal and collaborators designed poly (lactic-*co*-glycolic acid) Tam NPs that showed increased bioavailability and a milder effect on body weight [44]. Our in vivo homing results show selective targeting of iRGD-PS-Tam to the tumor and very little accumulation in non-malignant tissues. Moreover, adjacent normal mammary tissue did not uptake the iRGD-labelled PS. In-depth therapy studies are required to test whether improved biodistribution of iRGD-targeted TAM-PS translates into improved efficacy/side effects profile and whether this could be a therapeutic alternative that leads to the elimination of metastatic dormant cells.

About half of patients will develop resistance either de novo, or after several years of treatment (acquired resistance) [5]. In this sense, the development of treatments that target more than one signaling pathway is of great interest for breast cancer patients. Accumulating evidence suggests that the tumor microenvironment is involved in the development of resistance to conventional treatments and that patient therapy should be determined according to the specific tumor microenvironment constitution. Integrin β1 has been associated to treatment resistance in various tumor types and in the context of different treatment strategies, such as radiotherapy and chemotherapy [45–48]. We and others have shown that signaling of FN through integrin β1 is associated to endocrine resistance in breast cancer [6, 49]. In this context, we hypothesized that blocking integrin β1 together with ER would be a rational approach to improve the impact of Tam. Our results show that in 2D cultures on BSA the cytotoxic activity of iRGD-PS-Tam is similar to free Tam for both MCF7 and T47D cells. However, when seeded on FN, sensitivity to the anti-estrogen is recovered when the cells are treated with iRGD-PS-Tam. Of critical importance is the demonstration here that Tam contained within the iRGD-PS-Tam effectively interfered with ER’s transcriptional activity, the key driver of tumor progression in ER+ breast cancers. To our knowledge this is the first demonstration of Tam delivered in a nanovehicle actually acting at the transcriptional level. A previous effort reported by Vural and collaborators carried out transcription reporter assays but were not able to demonstrate inhibition of ER’s transcriptional activity by Tam vehiculized in β-cyclodextrin NPs [50]. Other authors have been able to show inhibition of ERs transcriptional activity using RU 58668, a selective ER downregulator, by encapsulating it in PEG-PLA nanospheres [51]. TAM and RU 58668 have different mechanisms of action [52] and thus delivery strategies may determine how they function at the molecular level.

β1 integrin is not only associated to metastasis and drug resistance, but additionally to stemness in the mammary gland [53, 54]. Thus, we asked the question of whether iRGD would interfere with the stem-cell enrichment effect that has been previously associated to Tam [9, 55]. We found that, contrary to what we had previously observed in the presence of Tam, iRGD-PS-Tam reduced the number of cells with self-renewing capacity. Interestingly, adding iRGD to free Tam had the same effect. These results have important clinical implications for all types of breast cancer. First-line chemo and radiotherapy have been shown to lead to a selection of breast cancer stem cells that are more resistant to apoptosis [56–58]. A study using HepG2 liver cancer cells showed that iRGD increased the impact of salinomycin nanomicelles on the population of cancer cells with self-renewing capacity as evaluated by suspension sphere formation assays [59]. Further studies are needed to characterize the impact of iRGD on breast cancer stem cells and determined whether it may counteract their enrichment in the context of chemo and radiotherapy. However, our results together with those using HepG2 cells suggest iRGD may be especially useful in cancers that show a hierarchical relationship between cell populations within tumors [60, 61].

In summary, we show that iRGD directed Tam-loaded PS could be a potentially valuable tool to treat ER+ breast cancer. Further in vivo efficacy studies are underway, with a special emphasis in understanding the impact of this treatment, as compared to free Tam, on the population of cancer stem cells, and the establishment of metastatic foci in other organs.

## Methods

### Reagents

Tam (tamoxifen base) was purchased from Sigma-Aldrich, Germany. Phosphate-buffered saline (PBS) was purchased from Lonza, Belgium. Dulbecco's modified Eagle's medium (DMEM)/F12 was purchased from Sigma-Aldrich, Germany. MCF7 and T47D cell lines were purchased from ATCC. Athymic nude mice were purchased from HSD, and Balb/c mice were purchased from Charles River. Fluorescein (FAM) with a cysteine residue for PS coupling (FAM-Cys) was purchased from TAG Copenhagen A/S (Copenhagen, Denmark). iRGD peptide with an extra cysteine residue for PS coupling and labeled with fluorescein (FAM-Cys-iRGD; sequence:FAM-Cys-Ahx-CRGDKGPDC; Ahx = aminohexanoic acid) was synthesized at Sanford Burnham Prebys Medical Discovery Institute. Maleimide-PEG(5 kDa)-b-PCL(10 kDa) polymer (Mal-PEG-PCL) and PEG(5 kDa)-b-PCL(10 kDa) (PEG-PCL) were purchased from Advanced Polymer Materials Inc. (Montreal, Canada). Amicon filters were purchased from Merk Millipore, Germany.

### Preparation and characterization of PS

To generate FAM-labeled PS (FAM-PS-Tam) or FAM-labeled iRGD-functionalized PS (iRGD-FAM-PS-Tam) with encapsulated Tam, 8 mg of PEG-PCL polymer were mixed with 2 mg of Mal-PEG-PCL polymer in acetone. To this solution, 50 µL of a 20 mg/mL Tam solution in acetone was added and acetone was evaporated with a N_2_ flow to allow the formation of the polymer film. The films were hydrated with 1 mL of PBS and sonicated for 5 min. A solution of 0.4 mg of FAM-Cys or FAM-Cys-iRGD peptides in 100 µL of PBS were added to the mix and sonicated for 15 min.

For PS purification, the PS sample was centrifuged for 1 min at 500* g* and the supernatant was purified using Amicon filters with a molecular weight cut-off of 100 KDa. The volume of the final PS solution was 1 mL at a concentration of 10 mg polymer/mL. Dynamic Light Scattering (DLS) (Zetasizer Nano ZS, Malvern Instruments) was used to assess the PS polydispersity and average size. In addition, transmission electron microscopy (TEM) was used to visualize the PS surface topology, size, and structure [62, 63]. Briefly, PS in PBS were deposited onto copper grids at 1 mg/mL, stained with 0.75% phosphotungstic acid (pH 7), air-dried, and imaged by TEM (Tecnai 10, Philips, Netherlands). The amount of encapsulated Tam was quantified by ultra-performance liquid chromatography (UPLC) (Waters), using free Tam dissolved in MeOH to prepare the standard curve. Twenty five microliter of PS-Tam were mixed with 25 μL of MeOH and 5 μL of this mixture was run at 35 °C using water/acetonitrile with 0.1% formic acid as eluent and Acquity Ultraperformance UPLC BEH C18 1.7 μM 2.1 × 50 mm column. FAM-iRGD peptide on PS was measured with an Infinite M200 microplate reader (Tecan, Männedorf, Switzerland) using 96 well polystyrene assay plates (Corning Inc., Corning, USA) and excitation and emission wavelengths of 494 and 518 nm, respectively. To calculate the amount of FAM-iRGD peptides per mg of polymer, linear standards of the protein (0–50 µg/mL) were prepared to account for absorbance at 494 and 518 nm.

### Cell culture

The MCF7 [25], T47D [26] and HC11 [39] cell lines were routinely maintained in growth medium consisting of Dulbecco's modified Eagle's medium (DMEM/F12) supplemented with 10% fetal calf serum (FCS; GenSA, Buenos Aires, Argentina) and gentamicin in a humidified 5% CO_2_/air atmosphere. Serial passages were conducted by treatment of 80% confluent monolayers with 0.25% trypsin (Invitrogen, Carlsbad, CA) and 0.02% EDTA in Ca^2+^-free and Mg^2+^-free phosphate buffered saline (PBS).

### Cell viability assay

To evaluate the effects of FN, 24-well plates were coated by incubating with a solution of 2 µg/mL of FN or BSA for 1 h and washed with PBS. 6 × 10^4^ MCF7 or T47D cells were seeded per well in complete growth medium. The next day, cells were washed twice with PBS and incubated in phenol red free DMEM/F12 with 1% charcoal-stripped FCS (chsFCS).

To compare the effects of Tam encapsulated in PS with treatment with free Tam, cells were treated with: EtOH as a control, free Tam, PS-Tam, iRGD-PS-Tam, free iRGD, free Tam + free iRGD, PS-Tam + iRGD, and empty PS (PS). In all cases, working concentration of Tam was 5 × 10^–6^ M. The amount of iRGD in PS was calculated as mentioned before and the same amount was used as free iRGD. Cells were treated for 96 h, and counted using a Neubauer Chamber.

### Tumor spheroid generation

To generate spheroids, we adapted the hanging drop method [64]. Briefly, 1 × 10^4^ cells were seeded on the cover of 48-well plates in 20 μL drops. Covers were then inverted and incubated for 72 h until spheroids were fully formed, after which they were transferred into individual wells containing 500 μL complete medium coated with 1.5% agarose. Spheroids were fed every other day by carefully aspirating 250 μL of medium and replacing it with the same volume of fresh complete medium.

### Evaluation of cellular and spheroid uptake

MCF7, T47D and HC11 cells (5 × 10^5^ cells) were seeded on glass coverslips in a 24-well plate. After 24 h, PS were added to the cells at a concentration of 0.5 mg/mL and incubated for 1 h or 24 h. The spheroids were grown for 7 days after which PS were added at the same concentration as cell uptake. The cells or spheroids were washed with PBS, fixed with 4% of paraformaldehyde in PBS, immuno-stained with Alexa 488-conjugated goat anti-rabbit IgG antibody (Abcam, USA), stained with propidium iodide, mounted and imaged with fluorescence confocal microscopy (Zeiss LSM 510). The images were processed and analyzed using the Image J software.

### Mammosphere assays

Single cell suspensions derived from MCF7 or T47D cell lines were plated in 6-well low-attachment suspension culture plates (Greiner Bio-One, Koln, Germany) at a density of 1 × 10^4^ viable cells/mL. Cells were grown in 2 mL serum-free media supplemented with B27 (Gemini Bioproducts, West Sacramento, CA) and 20 ng/mL epidermal growth factor as previously described [9]. Mammospheres were counted after 5–8 days in culture using a Nikon Eclipse TE2000-S inverted microscope.

### Luciferase reporter transcription assay

MCF7 and T47D cells (1 × 10^5^ cells) were seeded in 48 well plate coated with BSA or FN (2 μg/cm^2^). The next day, cells were washed twice with PBS and then treated in phenol red free DMEM/F12 with 1% charcoal stripped FCS (chsFCS) for 24 h. Next, cells were transfected with PTK-ERE-Luc and pTK Renilla vectors using Lipofectamine 2000 (Sigma-Aldrich, St. Louis, MO) according to manufacturer instructions. The weight ratio of Lipofectamine reagent to DNA was 3:1. The next day cells were treated with estradiol (10^–8^ M), free Tam (10^–6^ M), free Tam + estradiol, iRGD-PS-Tam (10^–6^ M) and iRGD-PS-Tam + estradiol using EtOH as control. After a 20 h incubation, cells were harvested with 30 µL cell lysis buffer (Promega) and the firefly and renilla luciferase activities were determined using a dual luciferase assay kit (Promega) by measuring luminescence with a Wallac Micro-Beta scintillation counter (PerkinElmer Life Sciences). Firefly luciferase reporter activity was normalized to the renilla luciferase activity.

### In vivo biodistribution studies

Animal care was in accordance with institutional guidelines. Tumor models were induced according to protocols approved by the Estonian Ministry of Agriculture, Committee of Animal Experimentation (permit #48). Nude mice carrying 2.5 mg silastic estrogen pellets were injected in the right flank with 5 × 10^6^ MCF7 cells diluted in 100 μL of a solution 1:1 of DMEM F12 and Matrigel (Corning Inc., Corning, USA). The MCF7 tumors were grown for 3 weeks and the FAM-labeled PS were injected IV (1 mg of polymer in 100 μL of PBS) and 4 h later the animals were perfused with 10 mL of PBS. Experiments with the M05 syngeneic mouse mammary tumors were approved by the “Angel H. Roffo Institute” Animal Care Committee and carried in the Roffo Animal Facility, in Buenos Aires. M05 tumor cells were inoculated with a trocar in the right flank and allowed to grow for 4 weeks as previously published [38]. FAM-labeled PS were injected IV (1 mg of polymer in 100 μL of PBS) and 4 h later the animals were perfused with 10 mL of PBS. Tissues were snap-frozen in liquid nitrogen, and stored at − 80 °C. The excised tumors and organs were cryosectioned at 10 μm, fixed with 4% of paraformaldehyde in PBS, and immunostained with rabbit anti-fluorescein (Life Technologies, USA), rat anti-mouse CD31 (BD Biosciences, USA), or rabbit anti-neuropilin-1 (Santa Cruz Biotechnology) as primary antibodies, and with Alexa 488-conjugated goat anti-rabbit IgG and Alexa 647-conjugated goat anti-rat IgG (Invitrogen, USA) as secondary antibodies. The nuclei of cells were stained with 10 μg/mL DAPI or Red Dot Far Red Nuclear Stain (Biotium). Confocal images of the tissue sections were analyzed with image J software.

### Statistical analysis

The statistical significance of differences between groups was calculated by applying one-way ANOVA, followed by Bonferroni–Sidak’s multiple comparisons test. A value of p < 0.05 was considered significant.

## Supplementary information

**Additional file 1: Figure S1.** iRGD PS labelling was determined by fluorescence. A calibration curve was performed with different concentrations of free iRGD, and iRGD concentration was obtained by linear regression. Four independent experiments were performed with similar results; mean: 0.0265 ± 0.0025 mg/mL. Assays were run at 25 °C and a pH of 7.4.

## Data Availability

The datasets used and/or analyzed during the current study are available from the corresponding author on reasonable request.
