# Supplementary figures and images for: iRGD-guided tamoxifen polymersomes inhibit estrogen receptor transcriptional activity and decrease the number of breast cancer cells with self-renewing capacity
Source: J Nanobiotechnology. 2019 Dec 7;17:120. doi: 10.1186/s12951-019-0553-4 (PMC6898937; doi:10.1186/s12951-019-0553-4)

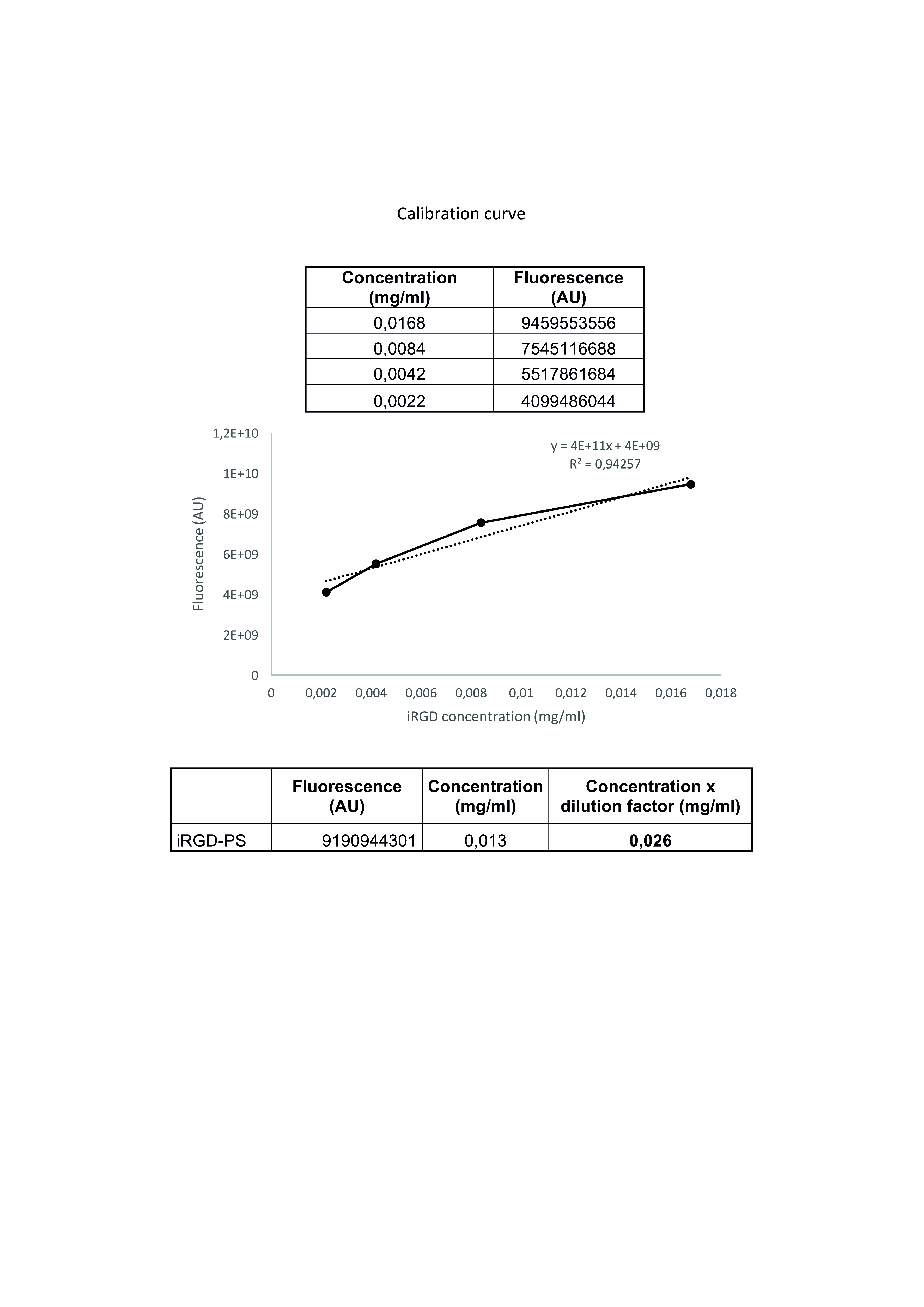

Supplement: Supplementary file 1 — Additional file 1: Figure S1. iRGD PS labelling was determined by fluorescence. A calibration curve was performed with different concentrations of free iRGD, and iRGD concentration was obtained by linear regression. Four independent experiments were performed with similar results; mean: 0.0265 ± 0.0025 mg/mL. Assays were run at 25 °C and a pH of 7.4. [file 12951_2019_553_MOESM1_ESM.tif]
